# Large Conization and Laparoendoscopic Single-Port Pelvic Lymphadenectomy in Early-Stage Cervical Cancer for Fertility Preservation

**DOI:** 10.1155/2013/207191

**Published:** 2013-10-07

**Authors:** Polat Dursun, Mete Caglar, Huseyin Akilli, Ali Ayhan

**Affiliations:** ^1^Division of Gynecologic Oncology, Department of Obstetrics & Gynecology, Baskent University School of Medicine, Kubilay Sokak No. 36, Maltepe, 06100 Ankara, Turkey; ^2^Duzce University School of Medicine, Duzce, Turkey

## Abstract

Fertility preservation in early-stage cervical cancer is a hot topic in gynecologic oncology. Although radical vaginal trachelectomy (RVT) is suggested as a fertility preserving approach, there are some serious concerns like cervical stenosis, second trimester loss, preterm delivery in survivors, and lack of residual tumor in the majority of the surgical specimens. Therefore, less radical surgical operations have been proposed in early-stage cervical carcinomas. On the other hand, single-incision laparoscopic surgery (SILS) is an evolving endoscopic approach for minimal access surgery. In this report, we present a case with early-stage cervical cancer who wishes to preserve fertility. We successfully performed single-port pelvic lymphadenectomy and large conization to preserve fertility potential of the patient. We think that combination of less radical approach like conization and single-port pelvic lymphadenectomy might be less minimally invasive and is still an effective surgical approach in well-selected cases with cervical carcinomas. Incorporation of single-port laparoscopy into the minimally invasive fertility sparing management of the cervical cancer will improve patients outcome with less complications and better cosmesis. Further studies are needed to reach a clear conclusion.

## 1. Introduction 

Carcinoma of the cervix is the second most common cancer among women and is also one of the leading causes of cancer-related deaths in females in both developed and developing countries. In developed countries, it accounts for 3.6% of new cancers, with an incidence of 14 cases per 100,000 women. The classical surgical management of early-stage cervical carcinoma requires the extirpation of the uterus and cervix, along with radical resection of the parametrial tissues and upper vagina, and this surgical approach, known as radical hysterectomy (RH), was described by Wertheim more than one hundred years ago. It is estimated that 43% of all cases of cervical carcinoma in the USA were diagnosed in women younger than 45 years of age in 2004. Additionally, 10%–15% of cervical cancer cases are diagnosed during the childbearing years as a result of some women waiting until their mid-to-late thirties to have children [1, 2]. Fertility preserving approach in cervical carcinoma has been gaining more and more interest around the world. The first successful systematic conservative surgical approach for invasive cervical carcinoma was described and published by Dargent in 1994 [1, 3]. Then, various surgical procedures like laparoscopic trachelectomy, radical abdominal trachelectomy, and simple vaginal trachelectomy with lymph node dissection and neoadjuvant chemotherapy plus trachelectomy have been described to preserve fertility in patients with cervical carcinoma [3–5]. More recently, less radical operations like large conization plus pelvic lymphadenectomy have been suggested instead of radical trachelectomy for fertility preservation in early-stage cervical carcinoma [4, 5].

On the other hand, single-port access surgery (SPAS), also known as laparoendoscopic single-site surgery (LESS) and single-incision laparoscopic surgery (SILS), is an evolving endoscopic approach for minimal access surgery. It was recently reported that single-port approach can be successfully used for benign and some malignant gynecologic conditions [6]. 

In this case, we first report a combined large conization plus laparoendoscopic pelvic lymphadenectomy as a fertility preserving approach for early-stage cervical carcinoma.

## 2. Case History 

### 2.1. Patient History

A 34-year-old woman, gravida 1, para 1, presented with the suspicion of the cervical carcinoma. Her routine pap test was reported as high-grade squamous intraepithelial lesion (HSIL) with suspicion of invasive disease. LEEP conization was performed and reported as microinvasive carcinoma with positive LVSI (stage Ia2). Rectovaginal examination under general anesthesia was normal. Abdominopelvic MRI investigation was performed and reported as suspicious of minimal residual disease in the endocervical canal and there were no lymph nodes metastasis and parametrial involvement. Due to strong fertility concerns of the patient, large conization and pelvic lymphadenectomy were performed.

### 2.2. Surgical Technique

The patient was placed in the modified lithotomy position under general anesthesia. Initially, the surgeon stood on the left side of each patient. The lateral sides of the umbilicus were everted using 2 clamps. Then, a 1.5–2 cm vertical intraumbilical skin incision was made. Sharp and blunt dissection was performed on the subcutaneous fatty tissue, the fascia was exposed and cut using a number 11 scalpel blade, and the peritoneum was incised using Metzenbaum scissors. The incision was then extended by an additional 0.5 cm via stretching of the skin. No other extraumbilical skin incisions were used.

**Figure 1:**
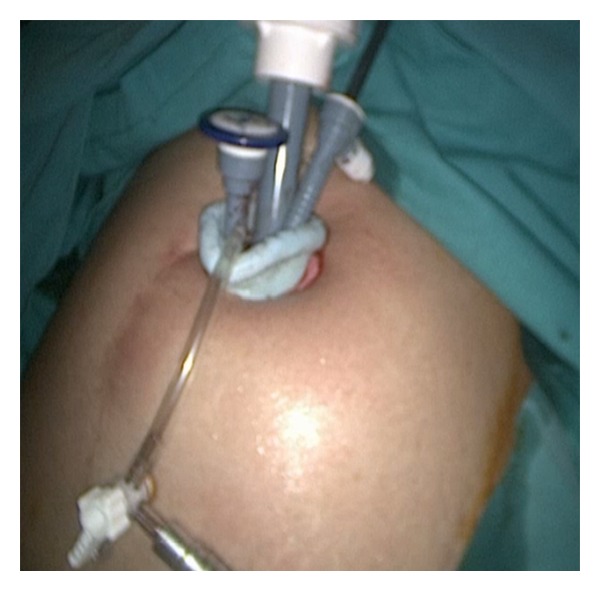
Appearance of SILS port and trocars at the beginning of the operation.

An SILS port (Covidien, Norwalk, CT, USA) with 3 access inlets was inserted into the abdominal cavity using a Heaney clamp, and a carbon dioxide pneumoperitoneum was created (Figure 1). A 10 mm rigid video laparoscope was used together with 2 classical nonroticulating straight laparoscopic instruments. One bipolar and 1 monopolar cautery, a pair of dissection forceps, and suction-irrigation devices were used sequentially as indicated during surgery. If collision of the instruments resulted in inadequate surgical movement for dissection, cutting, or coagulation, the surgeon changed the placement of the instruments, changed his position from the lateral side of the patient to the patient's head, or changed the placement of the endoscope in order to perform the necessary movements.

**Figure 2:**
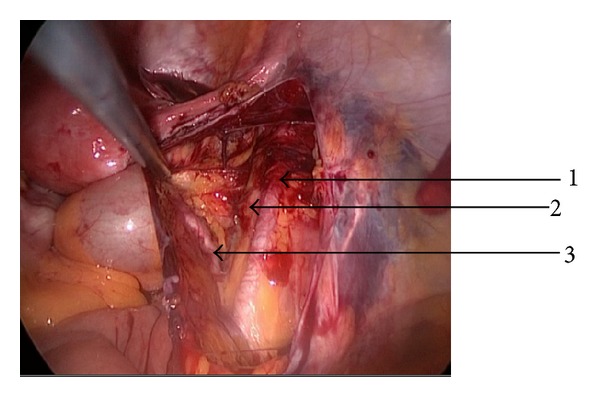
Retroperitoneal exposure and appearance of iliac vessels (1, 2), ureter (3).

**Figure 3:**
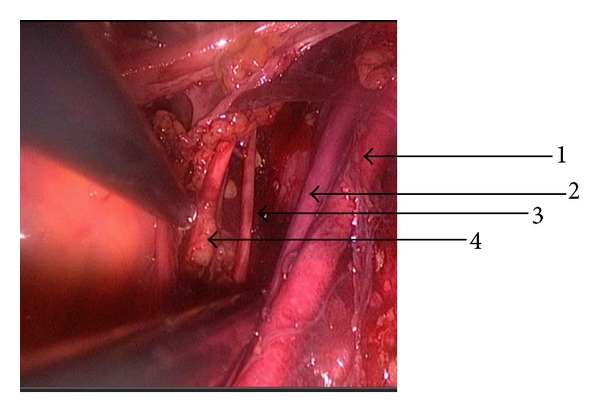
External iliac artery (1), external iliac vein (2), obturator nerve (3), and ureter (4) at the end of right pelvic lymphadenectomy.

First, right retroperitoneal area was exposed (Figure 2) by dissecting the overlying peritoneum on the iliopsoas muscle. Then, sharp and blunt dissection was performed to developed paravesical and pararectal fossae. Then, all the anatomical structures including external and internal iliac vessels, obturator fossa, and overlying lymph nodes were completely exposed (Figure 3).

**Figure 4:**
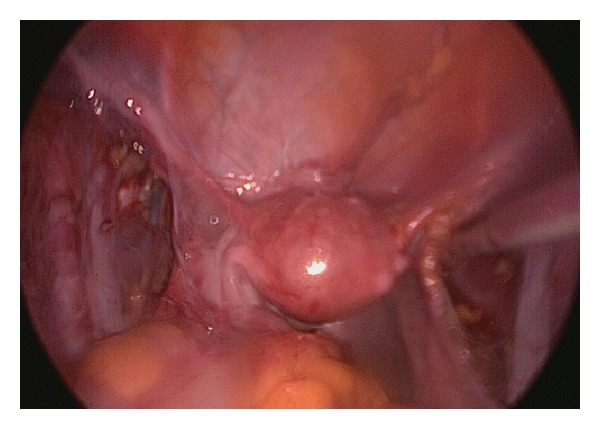
Final appearance of the external iliac vessels and uterus at the end of bilateral pelvic lymphadenectomy.

Iliac lymph node dissection was started from the caudal part and proceeds to the caudal part of the iliac vessels. Then, lymph nodes in the obturator fossa were resected. Sharp and blunt dissection were used as indicated (Figure 4).

Lymph specimens were retracted from the umbilical incision at the end of the surgery. Then, lymph nodes were sent to frozen section analysis. All the lymph nodes were reported as tumor free. Then, large conization was performed and conization material was also sent for frozen evaluation. Surgical margins of the conization specimen were also tumor negative. Operations were finished after homeostasis. The total operative time was 240 minutes, and the estimated blood loss was 150 mL. There were no intraoperative or postoperative major complications. The patient is still alive without any sign of the recurrence 8 months after the operation and still tries to conceive spontaneously.

## 3. Discussion 

Fertility preservation is one of the hot topics in gynecological cancers. Radical vaginal trachelectomy (RVT) was suggested as a fertility preserving approach in 1994, and today, it is accepted as a valid and oncologically safe surgical approach for early-stage cervical cancers. It has been reported that recurrence and death rates (4.2% and 2.8%, resp.) of RVT seem to be comparable to classical radical abdominal hysterectomy. On the other hand, RVT is very effective to preserve fertility in women with early-stage cervical cancer [1]. In a review of the RVT published in the literature, a 70% pregnancy rate was reported in the women who did attempt to conceive after RVT. However, 30% of these pregnancies were lost during the first and second trimesters. The rate of first trimester loss was 21%, while the second trimester loss was 8%. Furthermore, overall preterm deliveries (<36 weeks) occurred in 20% of these pregnancies [1, 7].

However, there are some serious concerns about RVT. Risk of second trimester loss and preterm delivery is one of the major drawbacks in women who became pregnant after an RVT. The second important complications are the cervical stenosis and related complications. Another important concern is the lack of residual tumors in the RVT specimens in 60% of the patients. Because of the aforementioned drawbacks of the RVT, some investigators suggested less radical trachelectomy like simple trachelectomy or large conization with or without lymphadenectomy in patients with small volume cervical cancers [4, 7, 8]. 

In order to reduce the risks and complications of RVT, total laparoscopic trachelectomy has been suggested as a minimally invasive approach; however, it is obvious that this might be more difficult than the classical RVT [9]. Rendón et al. reported that, to date, 44 cases of laparoscopic abdominal radical trachelectomy had been reported in 7 articles in the literature [10].

On the other hand, single-incision laparoscopic surgery (SILS) is accepted as the next frontier of minimally invasive gynecologic surgery and is currently in the initial stages of clinical use. There are growing interest and enthusiasm for SILS among surgeons and the medical industry [11, 12]. The first single-port appendectomy was performed in 2005, followed by the first single-port cholecystectomy in 2007. Today, complex urological, gynecological, colorectal, and bariatric surgical procedures have been performed using the SILS technique and equipment. Use of SILS has been facilitated by the introduction of rotating and curved instruments into clinical practice [11–13].

Although there have been several reports of SPA laparoscopy utilized to treat benign gynecologic disorders, single-port laparoscopic pelvic lymph node dissection was reported in a few reports in the literature. Fader and Escobar reported 12 patients by laparoendoscopic single-site surgery for the treatment of various gynecologic oncology conditions first time in 2009 and they concluded that SILS was feasible in selected patients with gynaecologic cancers [14]. The same group updated their experience with single-port laparoscopic surgery in 21 patients with gynecological cancers. Median overall operating time was 120 minutes (range, 60–185 minutes). Median pelvic and para-aortic node counts were 14 and 6, respectively. The authors concluded that the technique was feasible, and no morbidity was noted [15].

Usage of single-port approach has been also pioneered in locally advanced cervical carcinomas to assess para-aortic metastasis via extraperitoneal para-aortic lymphadenectomy [16]. In 13 patients with locally advanced cervical carcinoma, Lambaudie et al. reported the feasibility of single-port surgery (SPS) for laparoscopic extraperitoneal aortic dissection. In this study, lymph node count was similar to that of conventional laparoscopic extraperitoneal dissection [17]. Escobar et al. compared single-port laparoscopy (SPL), standard laparoscopy, and robotic surgery in patients with endometrial cancer. There were no significant differences in median operating time or estimated blood loss between the 3 groups. The median number of pelvic lymph nodes obtained was significantly higher in the robotic and SPL group compared with the laparoscopy group. Finally, the authors concluded that SPL surgery for endometrial carcinoma is feasible with similar operating times, hospital length of stay, complication rates, and estimated blood loss when compared with laparoscopy and robotics [18]. Single-port extrafascial hysterectomy and single-port radical hysterectomy with pelvic lymph node dissection were described in the gynecology literature [19, 20]. It has been reported that the risk of parametrial invasion and lymph node metastasis might be small in low volume cervical cancer. Therefore, in these clinical settings, less radical approaches like conization with or without lymphadenectomy might be appropriate as in our patient [21].

To the best of our knowledge, there are no reported studies about the combination of large conization and single-port pelvic lymphadenectomy in order to preserve fertility in early-stage cervical carcinoma in the literature. This approach minimizes the surgical trauma and may provide early ambulation and discharge with better cosmetic results. We also believe that incorporation of single-port laparoscopy into the minimally invasive fertility sparing management of the cervical cancer will improve patients outcome with less complications and better cosmesis. Further studies are needed to reach a clear conclusion about the efficacy, safety, and potential benefits of this technique in fertility preserving purposes in cervical cancers.
